# Quinomycin A targets Notch signaling pathway in pancreatic cancer stem cells

**DOI:** 10.18632/oncotarget.6560

**Published:** 2015-12-11

**Authors:** Sivapriya Ponnurangam, Prasad R. Dandawate, Animesh Dhar, Ossama W. Tawfik, Rajashri R. Parab, Prabhu Dutt Mishra, Prafull Ranadive, Rajiv Sharma, Girish Mahajan, Shahid Umar, Scott J. Weir, Aravind Sugumar, Roy A. Jensen, Subhash B. Padhye, Arun Balakrishnan, Shrikant Anant, Dharmalingam Subramaniam

**Affiliations:** ^1^ Department of Molecular and Integrative Physiology, The University of Kansas Medical Center, Kansas City, KS 66160, USA; ^2^ Department of Surgery, The University of Kansas Medical Center, Kansas City, KS 66160, USA; ^3^ Department of Cancer Biology, The University of Kansas Medical Center, Kansas City, KS 66160, USA; ^4^ Department of Pathology and Laboratory Medicine, The University of Kansas Medical Center, Kansas City, KS 66160, USA; ^5^ Department of Pharmacology, Toxicology and Therapeutics, The University of Kansas Medical Center, Kansas City, KS 66160, USA; ^6^ Department of Internal Medicine, The University of Kansas Medical Center, Kansas City, KS 66160, USA; ^7^ The University of Kansas Cancer Center, Kansas City, KS 66160, USA; ^8^ Piramal Life Sciences Inc, Goregaon East, Mumbai 400063, India; ^9^ Interdisciplinary Science and Technology Research Academy, Abeda Inamdar Senior College, Azam Campus, Pune, 411001, India

**Keywords:** DCLK1, apoptosis, tumor xenograft, Hes1, NICD

## Abstract

Cancer stem cells (CSCs) appear to explain many aspects of the neoplastic evolution of tumors and likely account for enhanced therapeutic resistance following treatment. Dysregulated Notch signaling, which affects CSCs plays an important role in pancreatic cancer progression. We have determined the ability of Quinomycin to inhibit CSCs and the Notch signaling pathway. Quinomycin treatment resulted in significant inhibition of proliferation and colony formation in pancreatic cancer cell lines, but not in normal pancreatic epithelial cells. Moreover, Quinomycin affected pancreatosphere formation. The compound also decreased the expression of CSC marker proteins DCLK1, CD44, CD24 and EPCAM. In addition, flow cytometry studies demonstrated that Quinomycin reduced the number of DCLK1+ cells. Furthermore, levels of Notch 1–4 receptors, their ligands Jagged1, Jagged2, DLL1, DLL3, DLL4 and the downstream target protein Hes-1 were reduced. The γ-secretase complex proteins, Presenilin 1, Nicastrin, Pen2, and APH-1, required for Notch activation also exhibited decreased expression. Ectopic expression of the Notch Intracellular Domain (NICD) partially rescued the cells from Quinomycin mediated growth suppression. To determine the effect of Quinomycin on tumor growth *in vivo*, nude mice carrying tumor xenografts were administered Quinomycin intraperitoneally every day for 21 days. Treatment with the compound significantly inhibited tumor xenograft growth, coupled with significant reduction in the expression of CSC markers and Notch signaling proteins. Together, these data suggest that Quinomycin is a potent inhibitor of pancreatic cancer that targets the stem cells by inhibiting Notch signaling proteins.

## INTRODUCTION

Pancreatic cancer is the fourth leading cause of adult cancer related death in USA with five-year survival rates at < 6%. In 2015, an estimated 40,560 Americans (20,710 men and 19,850 women) will die of the disease coupled with an additional 48,960 new cases (24,840 men and 24,120 women) [1]. By 2030, the disease is predicted to be the second leading cause of cancer related deaths [2]. Despite advances in molecular pathogenesis, pancreatic cancer remains a major unsolved health problem [3]. It is a rapidly invasive, metastatic tumor which is resistant to standard therapies [4]. At present, single agent based chemotherapy (e.g. Gemcitabine) is the mainstay treatment for metastatic adenocarcinoma of pancreas, but the tumor response rate is below 10%. Similarly none of the other current chemotherapeutic agents have an objective response rate of over 10% [3, 4]. The magnitude of this problem mandates the need for novel therapeutic agents.

**Figure 1:**
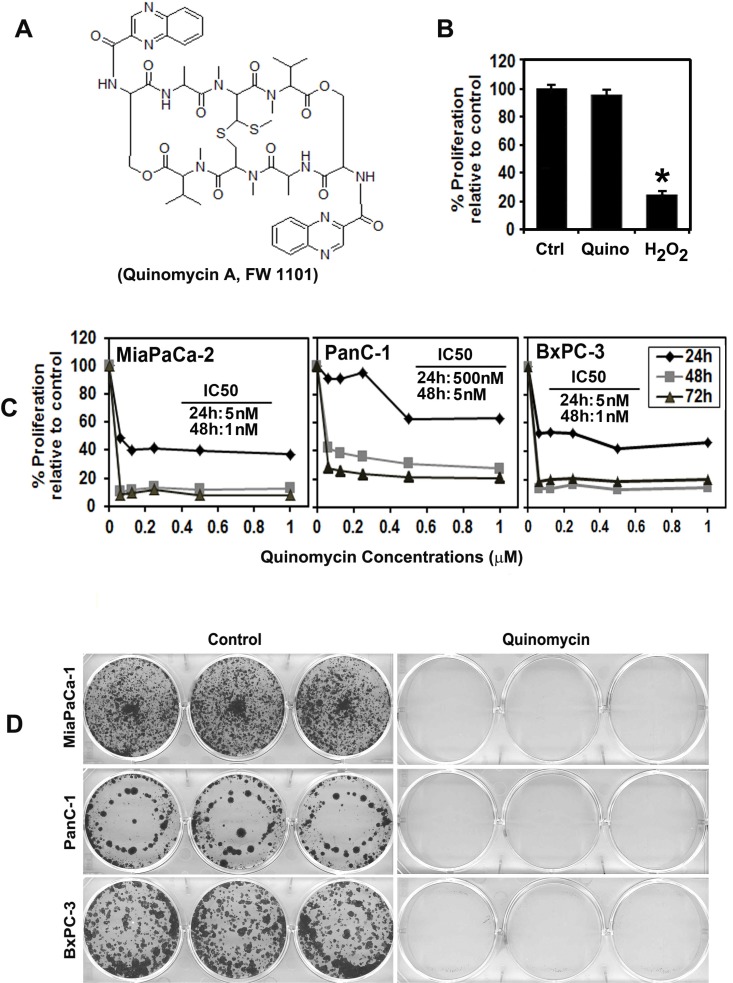
Quinomycin inhibits pancreatic cancer cell proliferation (**A**) Chemical structure of Quinomycin. (**B**) Proliferation of pancreatic ductal epithelial cells is not affected by 50 nM Quinomycin treatment for 48 h. (**C**) Quinomycin inhibits proliferation of pancreatic cancer cells. Cells were incubated with increasing doses of Quinomycin (0–1 μM) for up to 72 h and analyzed for cell proliferation. Quinomycin treatment resulted in a significant dose and time-dependent decrease in cell proliferation in all three cell lines when compared with untreated controls. (**D**) Quinomycin inhibits colony formation. Pancreatic cancer cells were incubated with 5 nM Quinomycin for 48 h and allowed to grow into colonies for 10 d. Incubation with Quinomycin inhibits colony formation. Results are representative of three independent experiments.

Quinomycin (also called Echinomycin, Figure 1) is a quinoxaline antibiotic that was originally isolated from *Streptomyces echinatus* [5]. Several studies have shown that it has antitumor activity with the ability to bifunctionally intercalate with double stranded DNA [5]. Quinomycin-induced apoptosis in HT-29 cells occurs via NF-κB activation by modulating IL-8 chemokine expression [6, 7]. In a mouse model of relapsed AML, low dose Quinomycin selectively targets leukemia-initiating cells and spares normal hematopoiesis [8]. Likewise, Quinomycin can be used to treat relapsed AML without affecting host normal hematopoietic stem cells. Moreover, National Cancer Institute sponsored phase II clinical trials has demonstrated anti-tumor efficacy of Quinomycin using various treatment schedules for various cancer types [9–19]. In addition, Quinomycin was shown to suppress leukemia cell growth in association with reduced Notch1 expression [20]. However, none of these studies were performed in pancreatic cancer patients.

Notch signaling plays a fundamental role in the differentiation and maintenance of stem cells. Aberrant activation of the Notch signaling has been associated with the development of many cancers, including pancreatic cancers [21, 22]. In fact, Notch signaling has been shown to play a contributing role in the development of pancreatic cancer [23, 24]. Furthermore, the pathway is deemed to be important in maintaining the cancer stem cell population in pancreatic cancer [25]. Interaction of Jagged-1 or Jagged-2 with the Notch-1 receptor promotes a γ-secretase-dependent cleavage of the receptor and release of the Notch intracellular domain (NICD), which translocates to the nucleus and activates transcription of Notch target genes such as Hes-1 and Hey1 [24]. Increased expression of Notch genes and their ligands has been detected in human pancreatic cancer tissues [24]. Overexpression of NICD accelerates the formation of oncogenic K-Ras-induced PanIN lesions [26]. Oral administration of γ-secretase inhibitor in mice blocks the progression of PanIN to ductal adenocarcinoma [27]. γ-secretase is a multiprotein intramembrane-cleaving protease with a growing list of protein substrates, including the Notch receptors. The four components of γ-secretase complex, Presenilin, Nicastrin, Pen2, and Aph1 are all thought to be essential for activity [24]. The catalytic domain resides within presenilin; nicastrin has been suggested to be critical for substrate recognition.

CSCs are the cells within a tumor that exclusively have self-renewal capacities, can give rise to all cancer cell lineages within a tumor, and are exclusively tumorigenic *in vivo*. They are able of undergoing asymmetric/symmetric cell division, can maintain and expand themselves and also have a distinct profile of surface marker expression that has been linked to poor prognosis [28]. Intriguingly, it has been shown that CSCs are highly resistant to standard therapy [29, 30]. CD44+CD24+EpCAM+ [31], CD133+ [32], ALDH+ are markers for prospectively identifying pancreatic cancer stem cells [33]. We have demonstrated that doublecortin and CaM kinase-like-1 (DCLK1) is an intestinal stem cell marker that is expressed in colon adenocarcinoma [34] and also in pancreatic adenocarcinoma [35]. Recent studies also demonstrated that DCLK1 distinguishes between tumor and normal stem cells in the intestine and could be a therapeutic target for colon cancer [36, 37]. Most recently, DCLK1 has been shown to mark a morphologically distinct subpopulation of cells with stem cell properties in pre-invasive pancreatic cancer [38]. In addition, DCLK1 expression was observed to be occur in early stage pancreatic cancer and in both early and late pancreatic intraepithelial neoplasia (PanIN) and that it increases as disease progresses in genetically engineered mouse models and also in human pancreatic cancer [26]. In this article, we have determined the effect of Quinomycin on pancreatic cancer stem cells and the Notch signaling pathway.

## RESULTS

### Quinomycin inhibits pancreatic cancer cell proliferation

Previous studies have shown that Quinomycin has antitumor activity with an ability to bifunctionally intercalate with double stranded DNA [5]. We first determined the effect of Quinomycin on proliferation of three pancreatic cancer cell lines, MiaPaCa-2, BxPC-3 and PanC-1 (Figure 1C). Quinomycin significantly suppressed the proliferation of these pancreatic cancer cells in a dose and time dependent manner. This anti-proliferation effect on tumor cells was seen within 24 h at a dose of 5 nM, which continued to significantly increase over the next 72 h (Figure 1C). In contrast, Quinomycin did not affect the proliferation of normal human pancreatic ductal epithelial cells (HPNE) even when treated at 50 nM (Figure 1B). As a positive control for cell death, we used hydrogen peroxide. These data suggest that Quinomycin is not toxic to normal cells. To determine the long-term effect of Quinomycin treatment, cells were treated with 5 nM Quinomycin for 48 h, following which the cells were allowed to grow in normal medium. Quinomycin treatment suppressed colony formation in all pancreatic cancer cell lines (Figure 1D), suggesting that Quinomycin-mediated effects on the tumor cells were irreversible.

### Quinomycin treatment induces PreG0/G1 arrest and apoptosis

**Figure 2:**
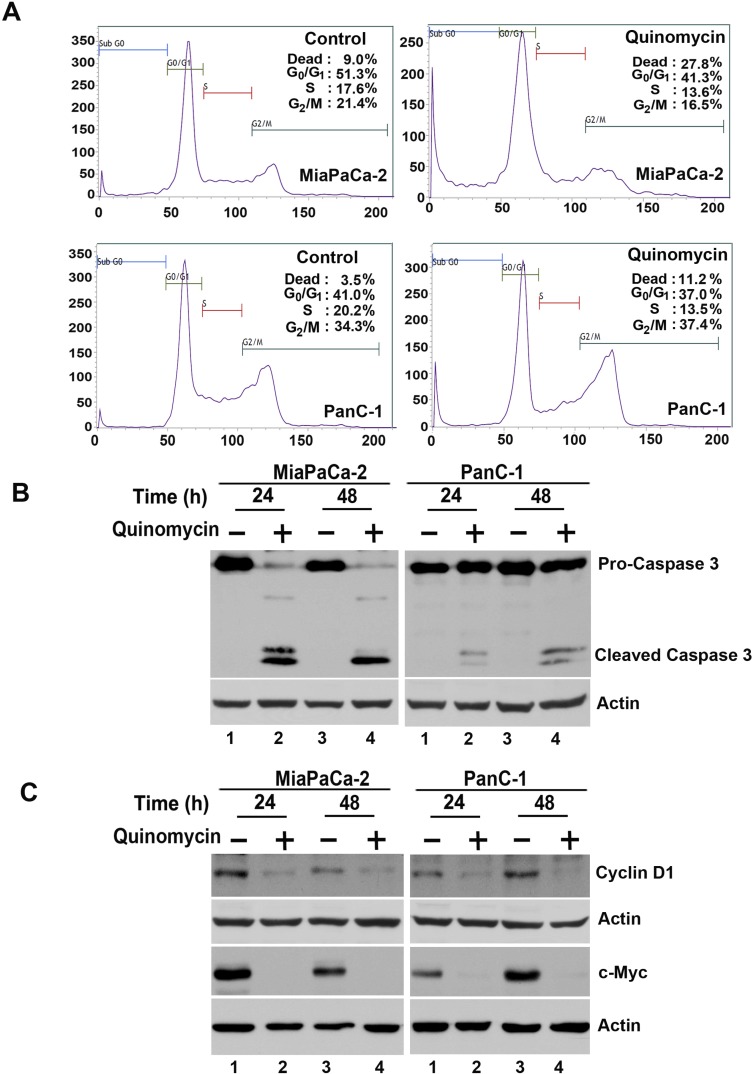
Quinomycin induces cancer cell apoptosis (**A**) Cell cycle analysis of Quinomycin treated cells. MiaPaCa-2 or PanC-1 cells were treated with up to 5 nM Quinomycin for 24 h and examined by flow cytometry following propidium iodide staining for DNA content. Quinomycin treatment leads to increased number of cells in the PreG0/G1 arrest. Graphs are representative of data collected from three experiments. (**B**) Quinomycin induces caspase 3, an apoptosis mediator. Lysates from MiaPaCa-2 or PanC-1 cells incubated with 5 nM Quinomycin were analyzed by western blotting for caspase 3 protein levels using rabbit anti-caspase 3 antibody. Quinomycin treated cells shows cleaved (activated) caspase 3 while untreated cells have no cleaved caspase-3. (**C**) Lysates from MiaPaCa-2 or PanC-1 cells incubated with 5 nM Quinomycin were analyzed by western blotting for cyclin D1 and c-Myc proteins. Both cyclin D1 and c-myc were reduced following Quinomycin treatment.

Given that Quinomycin inhibits proliferation and colony formation, we next determined whether Quinomycin affects cell cycle progression. Treatment with Quinomycin significantly induced Pre-G0/G1 arrest in both MiaPaCa-2 and PanC-1 cells (Figure 2A). Caspase-3 is key effector proteins in the apoptosis pathway [40]. Western blot analyses of MiaPaCa-2 and PanC-1 cell lysates showed a significant increase in activated caspase-3 in cells treated with 5 nM Quinomycin (Figure 2C). In addition, 5 nM Quinomycin inhibited the expression of cyclin D1 and c-Myc (Figure 2C). These data suggest that even at a dose of 5 nM, Quinomycin is a potent inducer of apoptosis of pancreatic cancer cells.

### Quinomycin inhibits pancosphere formation and expression of cancer stem cell marker proteins

**Figure 3:**
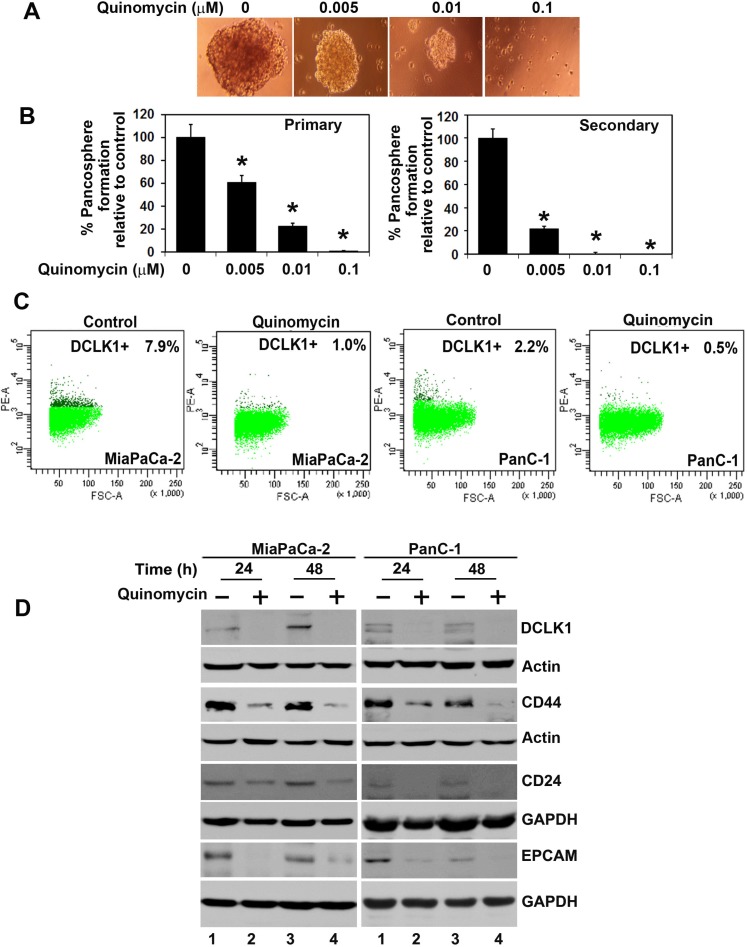
Quinomycin affects cancer stem cell marker expression (**A** and **B**) PanC-1 cells were grown in specific spheroid media in low adherent plates and treated with increasing concentrations of Quinomycin. After 5 days, the pancreatosphere were photographed and counted. The primary spheroids were collected and separated into single cells and replated. The Quinomycin treatment significantly was inhibited in both primary and secondary pancreatospheres formation (right and left panel)(*p < 0.05). (**C**) Sorting of anti-DCLK1 antibody -tagged phycoerythrin untreated MiaPaCa-2 and PanC-1 cells by flow cytometry. After 24 h, Quinomycin treatment caused significant reduction in the number of DCLK1 expressing cells. (**D**) Western blot analyses of lysates from Quinomycin treatment showed significant reduction in cancer stem cell marker DCLK1, CD44, CD24 and EPCAM protein levels in both MiaPaCa-2 and PanC-1 cells.

Defining the mechanisms that regulate stem cell fate is critical to increasing our understanding of the neoplastic process. Cancer stem cells are capable of self-renewal and generating tumors resembling the primary tumor [41]. Accordingly, we next determined the effect of Quinomycin on this population of cells. First, we determined the effect of Quinomycin on the ability to form pancreatosphere, a multicellular spheroid that is dependent on presence of stem cells to develop. Quinomycin treatment significantly inhibited PanC-1 pancreatosphere formation (Figure 3A and 3B). In addition, Quinomycin treatment further reduced secondary pancreatosphere (Figure 3B right panel). Flow cytometric analyses showed a significant decrease in DCLK1+ in both MiaPaCa-2 and PanC-1 cells following Quinomycin treatment (Figure 3C). Furthermore, Quinomycin treatment significantly inhibited expression of CSC markers DCLK1, CD44, CD24 and EPCAM in both MiaPaCa-2 and PanC-1 cells (Figure 3D). These data suggest that Quinomycin treatment affects pancreatosphere formation and CSC marker expression and especially DCLK1+ cells.

### Quinomycin inhibits Notch signaling by downregulating the γ-secretase complex

**Figure 4:**
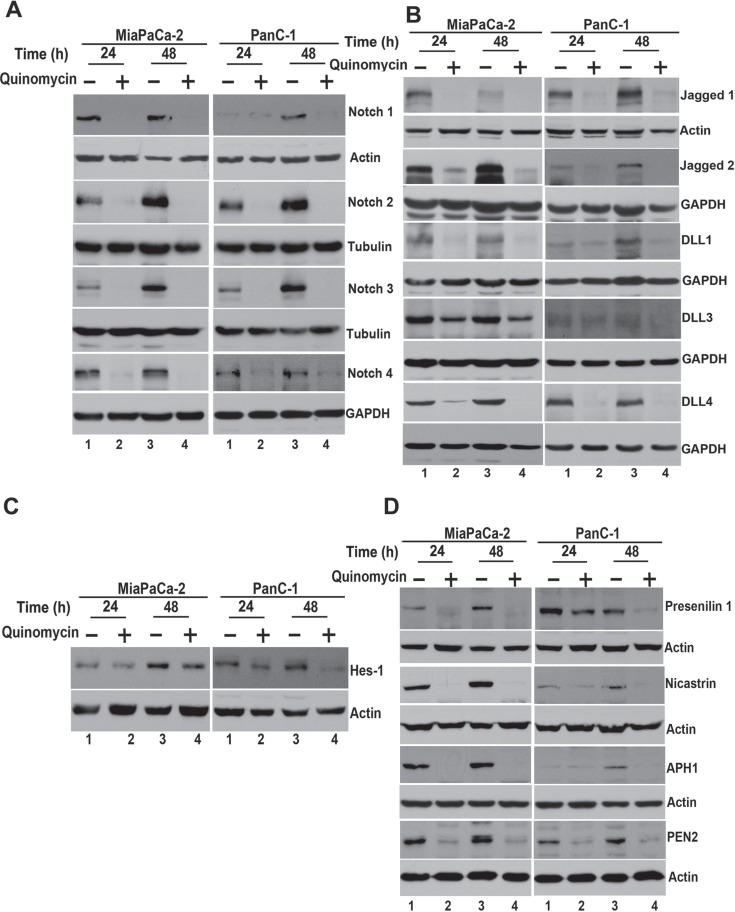
Quinomycin affects Notch signaling (**A**) Lysates from cells treated with Quinomycin caused significant reduction in the expression of Notch receptors Notch-1, 2, 3 and 4 in both MiaPaCa-2 and PanC-1 cells. (**B**) Lysates from cells treated with Quinomycin caused significant reduction in the expression of Notch ligands Jagged-1, 2 and Delta like ligand 1, 3 and 4 in both MiaPaCa-2 and PanC-1 cells. (**C**) Lysates from cells treated with Quinomycin caused significant reduction in the expression of Notch downstream target gene Hes-1 in both MiaPaCa-2 and PanC-1 cells. (**D**) Quinomycin also significantly reduced expression of γ-secretase complex proteins Presenilin-1, Nicastrin, APH1 and PEN2 in both MiaPaCa-2 and PanC-1 cells.

**Figure 5:**
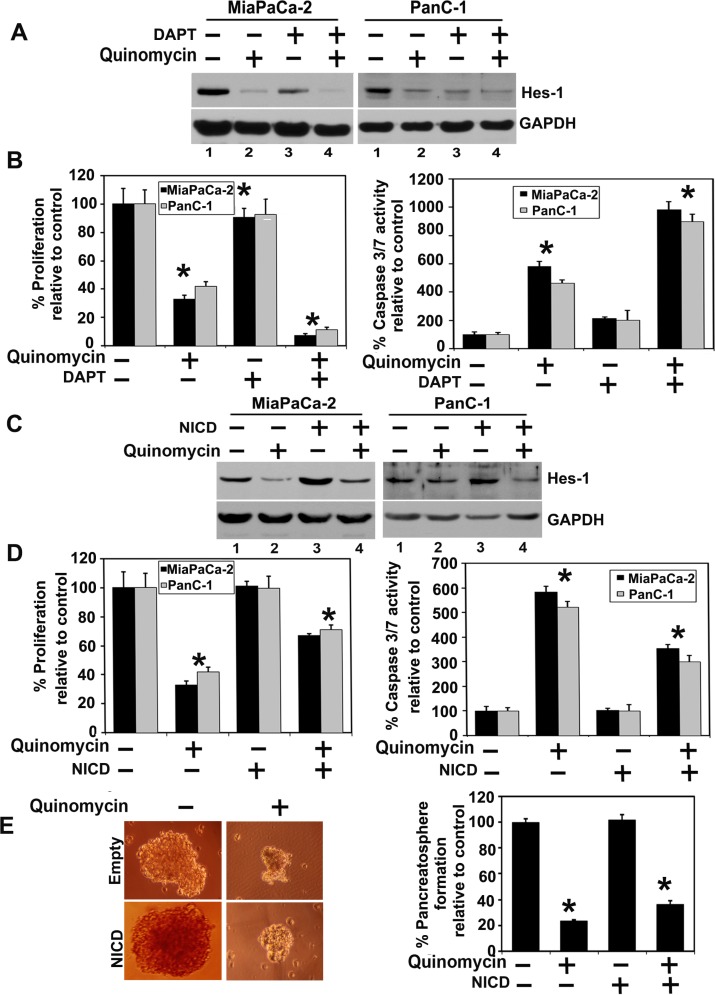
Quinomycin inhibits cell growth through inactivation of the γ-secretase complex (**A**) Cells were cotreated with Quinomycin in combination with γ- secretase complex inhibitor DAPT for 24 h. Lysates were analyzed by western blotting. The cotreatment of the Quinomycin in combination with γ- secretase complex inhibitor DAPT further reduced Hes-1 expression, (**B**) Cells were cotreated with Quinomycin in combination with γ-secretase complex inhibitor DAPT and subsequently measured for proliferation (left panel) and apoptosis (right panel). The cotreatment of the Quinomycin in combination with γ- secretase complex inhibitor DAPT further reduced proliferation (left panel) and further increased apoptosis (right panel). (**C**) Ectopic expression of NICD overcomes Quinomycin-mediated suppression of Hes-1 expression. Cells transiently expressing NICD was treated with Quinomycin for 24 h. Lysates were analyzed by western blotting. Hes-1 was increased in the NICD expressing cells when compared to vector transfected controls. (**D**) Cells expressing NICD were treated with Quinomycin and subsequently measured for proliferation (left panel) and apoptosis (right panel). Ectopic expression of NICD rescued Quinomycin mediated inhibition of cell proliferation and apoptosis (*P < 0.05). (**E**) Cells expressing NICD were treated with Quinomycin and subsequently performed for pancreatosphere formation (left and right panel). Ectopic expression of NICD rescued Quinomycin mediated inhibition of pancreatosphere formation (*P < 0.05).

We next determined the effect of Quinomycin on Notch signaling-related proteins in the pancreatic cancer cell lines. All four Notch receptors (Notch-1 to -4 were downregulated following Quinomycin treatment (Figure 4A). In addition, Notch ligands Jagged-1, 2 and Delta like ligand 1, 3 and 4 were downregulated following Quinomycin treatment (Figure 4B). Further confirmation was obtained when reduced expression of Hes-1 expression was observed (Figure 4C). We next determined whether the γ-secretase complex comprising of Presenilin, Nicastrin, APH1 and PEN2 was affected. Treatment with Quinomycin resulted in downregulation in the expression of all four proteins (Figure 4D). In addition, the co- treatment of Quinomycin in combination with γ-secretase complex inhibitor DAPT further reduced Hes- 1 expression (Figure 5A), and proliferation (left panel) while inducing apoptosis (right panel) (Figure 5B). These data suggest that Quinomycin-mediated downregulation of the Notch signaling pathway occurs at least in part through the inhibition of the γ-secretase complex.

Cyclin D1 overexpression has been linked to the development and progression of cancer [42]. In addition, a recent study demonstrated that cyclin D1 is a direct downstream target of the Notch signaling pathway [43]. Furthermore, c-Myc is upregulated in cancers and also a direct downstream target of the Notch signaling pathway [44, 45]. In both MiaPaCa-2 and PanC-1 cells, Quinomycin treatment resulted in reduced cyclin D1 and c-Myc expression (Figure 2C), suggesting that Quinomycin mediated downregulation of cyclin D1 and c-Myc occurs in part through the inhibition of the Notch signaling pathway.

### Quinomycin inhibits cell growth through inactivation of the γ-secretase complex

We next determined whether lack of Notch-1 activation is the reason for reduced growth of pancreatic cancer cells. For this, we expressed the intracellular domain NICD in MiaPaCa-2 and PanC-1 cells. Western blot analyses demonstrated increased expression of Hes-1 following ectopic expression of NICD in both cell lines (Figure 5C). More importantly, even in the presence of Quinomycin Hes-1 expression remained high in response to ectopic NICD overexpression. In addition, ectopic NICD overexpression reversed Quinomycin mediated inhibition of cell proliferation (Figure 5D). Futhermore, ectopic NICD overexpression resulted in significantly increased PanC-1 pancreatosphere formation, even in the presence of Quinomycin (Figure 5E left and right panel). Together, these data suggest that Quinomycin inhibits the γ-secretase complex thereby affecting Notch signaling.

### Quinomycin inhibits pancreatic tumor xenograft growth

**Figure 6:**
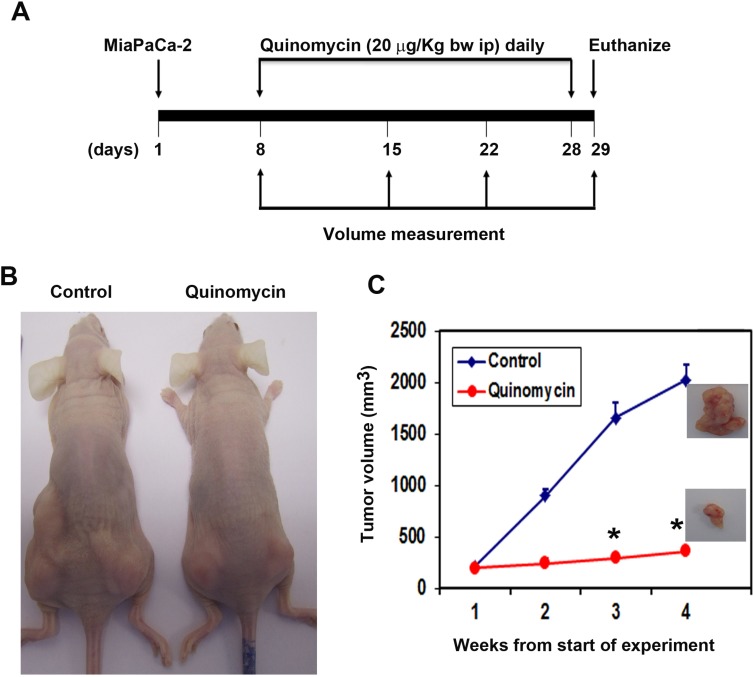
Quinomycin inhibits pancreatic cancer xenografts (**A**) MiaPaCa-2 cells were injected in to the flanks of nude mice and palpable tumors were allowed to develop for 7 days. Subsequently, Quinomycin (20 μg/kg bw) was injected daily intraperitoneally every day for 21 days. On day 22, tumors were excised and subject to further analyses. (**B**) Quinomycin treatment resulted in significantly lower tumor weight when compared to control. Tumor size was measured every week. There was a significant reduction in tumor size from Quinomycin treated animals when compared control (*P < 0.05). (**C**) Tumor volumes in Quinomycin treated mice were smaller when compared to control (*P < 0.05).

To evaluate the role of Quinomycin on tumor growth *in vivo*, we next examined its effects on growth of pancreatic cancer cell xenografts. MiaPaCa-2 pancreatic cancer xenograft tumors were allowed to develop and grow for one week following which Quinomycin (20 μg/kg bw) mixed with 5% sodium bicarbonate was administered intraperitoneally daily for three weeks. Quinomycin inhibited the growth of the tumor xenografts (Figure 6A and 6B). The excised tumors from control animals weighed ∼2000 mg, while those treated with Quinomycin weighed ∼300 mg (Figure 6C). In addition, tumor volume was significantly decreased (Figure 6C). There was no apparent change in liver, spleen, or body weight in the animals (data not shown). These data imply that Quinomycin is a potential therapeutic agent for treating pancreatic cancers but is relatively non-toxic to the animals.

### Quinomycin inhibits the expression of cancer stem cell markers and notch signaling proteins in tumor xenograft tissues

**Figure 7:**
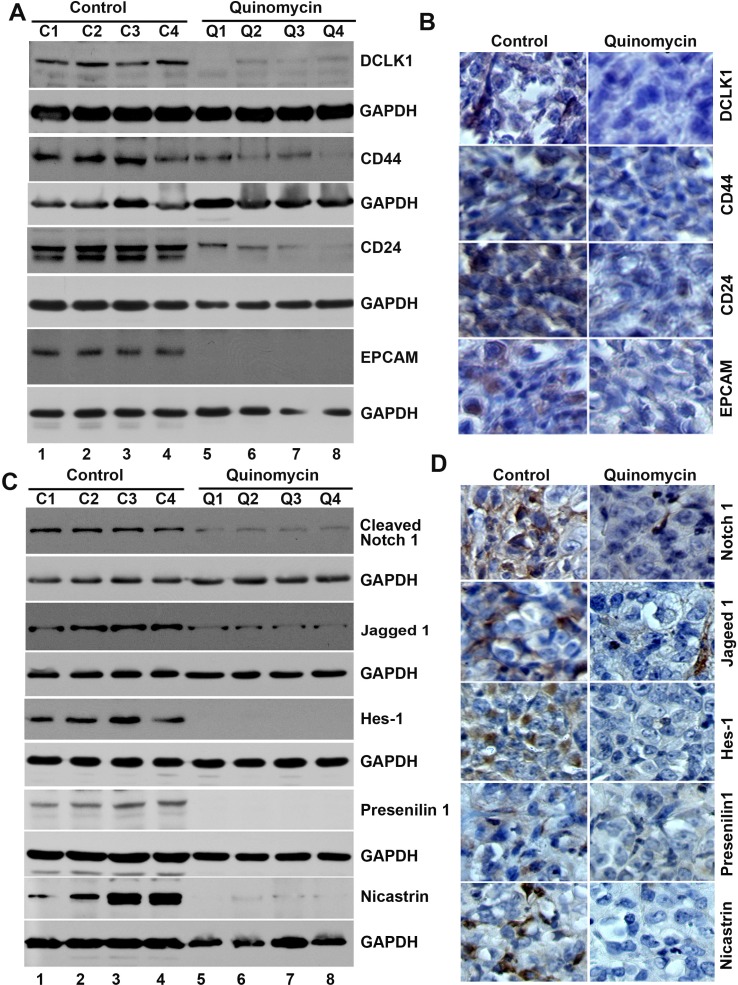
Quinomycin inhibit cancer stem cell marker proteins and Notch signaling in tumor xenografts (**A**) Western blot analysis showed that tissue lysates from Quinomycin treated animals have significantly lower levels of cancer stem cell markers. (**B**) Immunohistochemistry shows that treatment with Quinomycin significantly reduced the expression of cancer stem cell markers. (**C**) Western blot analysis showed that tissue lysates from Quinomycin treated animals have significantly lower levels of Notch-1, Jagged-1, Hes-1, and γ-secretase complex proteins. (**D**) Immunohistochemistry shows that Quinomycin treated animals have significantly lower levels of Notch-1, Jagged-1, Hes-1, and γ-secretase complex proteins Presenilin 1 and Nicastrin in the tumor xenograft tissues. (C1-C4: Controls, Q1-Q4: Quinomycin treated).

**Figure 8:**
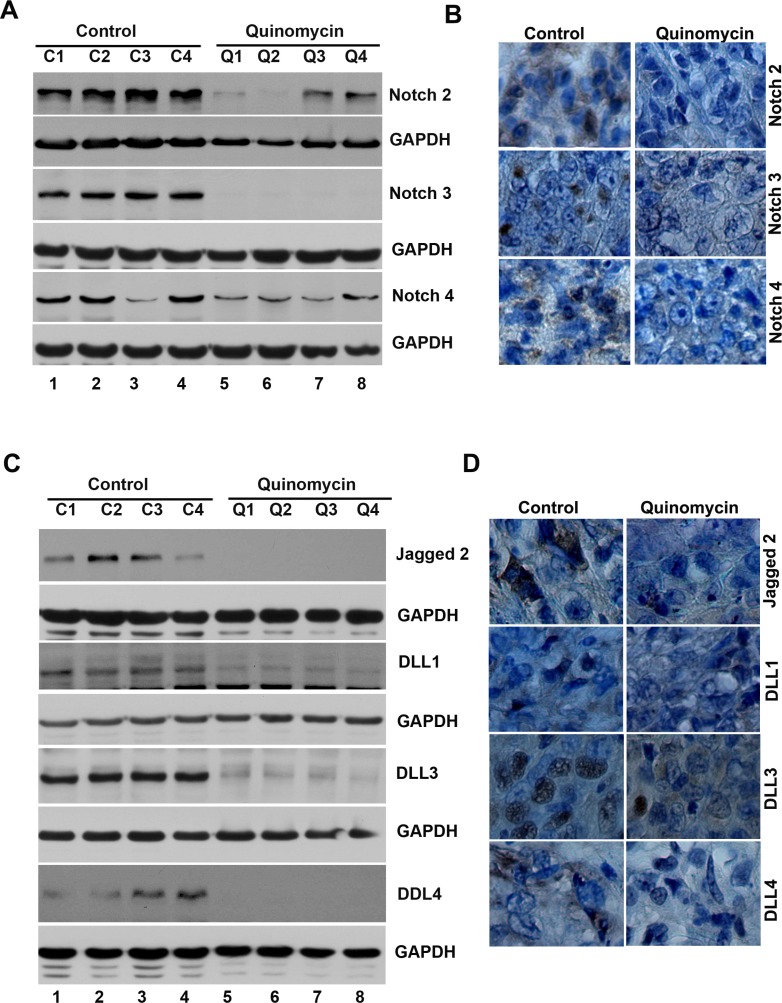
Quinomycin inhibit Notch receptor-2, 3 and 4 and its ligands Jagged 2, DLL-1, 3 and 4 in tumor xenografts (**A**) Western blot analysis showed that tissue lysates from Quinomycin treated animals have significantly lower levels of Notch-2, 3 and 4 receptor expression. (**B**) Immunohistochemistry shows that treatment with Quinomycin significantly reduced the expression of Notch-2, 3 and 4 receptors. (**C**) Western blot analyses showed that tissue lysates from Quinomycin treated animals have significantly lower levels of Notch ligand Jagged-2, DLL-1, 3 and 4 expressions. (**D**) Immunohistochemistry shows that treatment with Quinomycin significantly reduced the Notch ligand Jagged-2, DLL-1, 3 and 4 expressions. (C1–C4: Controls, Q1–Q4: Quinomycin treated).

To further investigate whether the Quinomycin affects CSCs, we determined specific marker expression in the tumor tissues. Western blot analyses demonstrated that Quinomycin treatment significantly reduced the expression of CSC proteins DCLK1, CD44, CD24 and EPCAM when compared to controls (Figure 7A). This was confirmed by immunohistochemistry (Figure 7B). These data suggest that Quinomycin targets pancreatic CSCs with high potency. Furthermore, we also examined the effects on Notch signaling in the tumor tissues obtained from control and Quinomycin treated mice. Quinomycin treatment resulted in significantly lower levels of activated Notch-1, its ligand Jagged 1 and the downstream target gene Hes-1 (Figure 7C). In addition, Quinomycin treatment resulted in significantly lower levels of Notch-2, 3, and 4 (Figure 8A) and its ligand Jagged 2, DLL-1, 3 and DLL4 (Figure 8C). This was confirmed by immunohistochemistry (Figure 8B and 8D). There was also a significant reduction in the expression of γ-secretase complex proteins, Presenilin 1 and Nicastrin (Figure 7C). Again, further confirmation of the downregulation was obtained by immunohistochemistry for the proteins in the xenograft tissue (Figure 7D). These data suggest that Quinomycin significantly affects the expression of Notch signaling-related proteins, which might contribute to the inhibitory effects of this treatment.

## DISCUSSION

We are the first to study and demonstrate that Quinomycin inhibits pancreatic cancer stem cells and the mechanism involves Notch signaling. Our results indicate that Quinomycin possesses great potential as a promising anti-pancreatic cancer therapeutic agent. Pancreatic cancer is one of the most lethal cancers and has emerged as a leading cause of cancer-related death in the western world, with most patients dying within one year of diagnosis. The significant morbidity, toxicity and poor response rates of current chemotherapy regimens have led to searches for less toxic alternative therapies. The data presented in the article show that Quinomycin inhibits the proliferation of pancreatic cancer cells, induces cell cycle arrest and apoptosis, resulting in reduced colony formation. These results were also replicated *in vivo*, where Quinomycin decreased tumor growth.

Recent studies have suggested that CSCs have the capacity to drive tumor recurrence and resistance to chemotherapeutic agents and radiation [46]. Natural compounds such as curcumin, sulforaphane and honokiol have been suggested to target CSCs [43, 47]. Our results suggest that the Quinomycin is a potent inhibitor CSCs. Markers for prospectively identifying pancreatic cancer stem cells are CD44+CD24+EpCAM+ [31], CD133+ [32], and ALDH+ [33]. We have demonstrated that DCLK1 is stem cell marker whose expression is upregulated in both colon and pancreatic adenocarcinomas [34, 35]. Recent studies demonstrated that DCLK1 distinguishes between tumor and normal stem cells in the intestine and could be a therapeutic target for colon cancer [36, 37]. Similarly, DCLK1 was shown to mark a morphologically distinct subpopulation of cells with stem cell properties in pre-invasive pancreatic cancer [38]. Here, we have demonstrated that Quinomycin inhibits DCLK1 expression. Moreover, we have demonstrated that Quinomycin also inhibits the expression of other stem cell marker proteins such as CD44, CD24 and EPCAM. This was also confirmed *in vivo* where the Quinomycin significantly reduced the expression of these markers and also the growth of the xenografts. Another method that is commonly used to demonstrate stemness is the growth of spheroids or pancreatospheres. Quinomycin inhibited pancreatosphere formation further suggesting that they target the CSCs.

The Notch pathway plays a critical role in pancreatic cancer. Notch also has been shown to be important in stem cell renewal and vascular development [48]. Notch 2-positive progenitors have the intrinsic ability to give rise to pancreatic ductal cells [49]. Notch3 and HEY-1 have been shown to be prognostic biomarkers in pancreatic adenocarcinoma [50]. Finally, Notch 4 is elevated in pancreatic adenocarcinoma [50]. A recent study demonstrated that this pathway is important in maintaining CSC population in pancreatic cancer [25]. In our studies, we have determined that Quinomycin resulted in downregulation of the Notch ligand Jagged1, 2 and DLL1, 3 and 4 as well as all four essential members of the γ-secretase complex, the critical enzyme that cleaves and releases the NICD from the membrane. Therefore, Quinomycin mediated inhibition of pancreatic cancer growth is partly mediated by inactivating Notch-1. This was further confirmed by the combination of a γ-secretase inhibitor with Quinomycin, which further inhibited proliferation and induced apoptosis. However, ectopic expression of NICD reversed the effects of Quinomycin and partially restored cell growth. It would also be interesting to determine whether there are other clients for the γ-secretase complex and the role of these client proteins in CSC biogenesis.

We believe there is a lot of promise for use of Quinomycin as a therapeutic and preventive agent for pancreatic cancer. It should be noted that the dose of Quinomycin demonstrating efficacy was quite low, because a dose of 20 μg/kg bw corresponds to a calculated human equivalent dose of 57 μg/m^2^. More importantly, at this dose, while there was significant efficacy against the xenograft tissue, there was no effect on normal tissues in the animals. This is really important an observation because previous human clinical trials have suggested that Quinomycin is toxic. However, the studies showing toxicity utilized doses from 1200 to 2128 μg/m^2^ administered IV [48]. At lower doses (60 and 120 μg/m^2^), there was no observed toxicity, with only grade I toxicity encountered at 180 μg/m^2^ [48]. Our IP dose equivalent of 57 μg/m^2^ is approximately 1/30th the lowest IV dose studies in solid tumor patients. Tumor growth inhibition at this dose level is also quite consistent with recent reports describing that IP doses of Quinomycin at a Human Equivalent IP Dose of 28.5 μg/m^2^ eliminated AML leukemia-initiating cells without a significant effect on the hematopoietic stem cells [8]. These studies provide a foundation for further clinical development of the drug for treatment of pancreatic cancers.

In conclusion, our current study provides evidence that treatment with Quinomycin results in growth inhibition *in vitro* and *in vivo*. We did not observe any significant toxicity in mice xenografts treated with Quinomycin. Furthermore, Quinomycin treatment was potent against pancreatic CSCs especially DCLK1+ cells. In addition, Quinomycin significantly suppressed Notch-receptor 1–4 activation through γ-secretase complex proteins. Taken together, these data suggest that Quinomycin targets pancreatic CSCs and is an attractive and potentially novel agent for the treatment and prevention of pancreatic cancer. However, it should be noted that while the compound is effective in inhibiting pancreatic cancers, this might not be universal. Hence, additional studies are essential to determine efficacy for different cancers.

## MATERIALS AND METHODS

### Cells and reagents

Human pancreatic cancer cells PanC-1, MiaPaCa-2, and BxPC-3 (all cell lines obtained from American Type Culture Collection, at passage 4) were grown in RPMI 1640 containing 10% heat inactivated fetal bovine serum (Sigma-Alrich, St. Louis, MO) and 1% antibiotic-antimycotic solution (Corning, Tewksbury, MA) at 37°C in a humidified atmosphere of 5% CO_2_. HPNE cells were kindly provided by Dr. Anirban Maitra, Johns Hopkins University School of Medicine and grown in DMEM with 4.5 g/L glucose, L-glutamine and Sodium Pyruvate (Corning, Tewksbury, MA) with 5% FBS, 1X N2, 10 ng/ml bFGF and 50 μg/ml Gentamycin. All the cell lines used in this study were within 20 passages after receipt or resuscitation (∼3 months of non-continuous culturing). The cell lines were not authenticated as they came from national repositories. Quinomycin was purchased from (AG Scientific, San Diego, CA) and our collaborator. N-[N-(3,5-Difluorophenacetyl)-L-alanyl]-S-phenylglycine t-butyl ester (DAPT) was purchased from (Sigma-Alrich, St. Louis, MO).

### Proliferation and apoptosis assays

To assess proliferation, cells were seeded on to 96 well plates and grown overnight before treatment with increasing doses of Quinomycin. Cell proliferation was determined by enzymatic hexoseaminidase assay as described previously [39]. For apoptosis, caspase 3/7 activity was measured using the Apo-one Homogeneous Caspase-3/7 Assay kit (Promega, Madison, WI).

### Clonogenicity assay

Briefly, 6 well dishes were seeded with 500 viable cells per well, treated with Quinomycin in 10% FBS containing RPMI1640 for 48 h, then medium containing compound was removed, and the cells were incubated for an additional 10 d in complete medium to allow colonies to form. The colonies were fixed in formalin, followed by staining with crystal violet. Experiments were done in triplicate.

### Cell cycle analyses

Cells were treated with Quinomycin for 24 h, and subsequently trypsinized and suspended in phosphate buffered saline (PBS). Single-cell suspensions were fixed using 70% ethanol for 2 h, and subsequently permeabilized with PBS containing 1 mg/ml propidium iodide (Sigma-Aldrich), 0.1% Triton X-100 (Sigma-Aldrich) and 2 μg DNase-free RNase (Sigma-Aldrich) at room temperature. Flow cytometry was done with a FACS Calibur analyzer (Becton Dickinson, Mountain, View, CA), capturing 10,000 events for each sample. Results were analyzed with ModFit LT^™^ software (Verity Software House, Topsham, ME).

### Western blot analysis

Cell lysates were subjected to polyacrylamide gel electrophoresis and blotted onto Immobilion polyvinylidene difluoride membranes (Millipore, Bedford, MA). Antibodies were purchased from Cell Signaling Technology (Beverly, MA), Abcam Inc. (Cambridge, MA), Sigma Aldrich, GenScript (Piscataway, NJ) and Santa Cruz Biotechnology Inc. (Santa Cruz, CA) and specific proteins were detected by the enhanced chemiluminescence system (GE Health Care, Piscataway, NJ).

### Flow cytometric analyses for CSC marker

24 h following Quinomycin treatment, cells were subjected to direct immunofluorescence staining followed by flow cytometric analyses. Briefly, the cells were harvested and suspended in PBS containing 0.5% BSA for 10 minutes at room temperature followed by the addition of 10 μl phycoerythrin conjugated DCLK1 antibody (Abcam Inc, Cambridge, MA). The samples were analyzed using a FACS Calibur analyzer (Becton Dickinson, Mountain, View, CA), capturing 10,000 events for each sample. Results were analyzed with ModFit LT^™^ software (Verity Software House, Topsham, ME).

### Pancreatosphere assay

Cells were cultured in RPMI 1640 supplemented with 20 ng/ml bFGF 10 mL per 500 mL of 50X B27 supplement, EGF 20 ng/ml (all from Life Technologies) at low densities (3000 cells/mL) in 6 well low adhesion plates. Cells were treated with Quinomycin. After 5 days, the number and size of pancreatospheres were determined using Celigo (Cyntellect Inc., San Diego, CA). For second and third passages, cells were grown in the absence of these compounds.

### Plasmids and transfections

MiaPaCa-2 and PanC-1 cells were transfected with plasmid EF.hICN1.CMV.GFP encoding the Notch-1 intracellular domain (NICD) or the empty vector EF.v-CMV.GFP (Addgene Inc, Cambridge, MA), and subsequently treated with 5 nM Quinomycin for 24 h and harvested for western blot analysis. Cell proliferation and apoptosis were detected using hexoaminadase assay and Apo-one Homogeneous Caspase-3/7 Assay kit, respectively. In addition, pancreatospheres assays were also performed.

### MiaPaCa-2 xenograft tumors in mice

Five-week-old male athymic nude mice, purchased from Charles River Laboratory were utilized for *in vivo* experiments. They were maintained with water and standard mouse chow ad libidum and used in protocols approved by the University's Animal Studies Committee. Animals were injected with 1 × 10^6^ MiaPaCa-2 cells in the left and right flank and allowed to form a xenograft. One week following implantation, and after observing the presence of a palpable tumor, Quinomycin (20 μg/kg body weight) was administered intraperitoneally daily for 21 d. Tumors were measured weekly. At the end of treatment the animals were euthanized, and the tumors were removed, weighed and use for histology (hematoxylin & eosin), immunohistochemistry, and gene expression studies.

### Immunohistochemistry

Paraffin embedded tissues were cut to 4 μm sections, deparaffinized and blocked with Avidin/Biotin for 20 min. The slides were incubated with primary antibodies for overnight at 4^°^C. Next the slides were treated with a broad spectrum secondary antibody (Invitrogen) and HRP-conjugate for one hour and then developed with DAB (Invitrogen). Finally, the slides were counterstained with hematoxylin. The slides were examined in Nikon Eclipse Ti microscope under a 40X objective.

### Statistical analysis

All values are expressed as the mean ± SEM. Data was analyzed using an unpaired 2-tailed *t* test. A *P* value of less than 0.05 was considered statistically significant.
